# Inhibition of the Glycolysis Prevents the Cerebral Infarction Progression Through Decreasing the Lactylation Levels of LCP1

**DOI:** 10.1007/s12033-022-00643-5

**Published:** 2022-12-27

**Authors:** Wei Zhang, Liang Xu, Zhenfei Yu, Meiqi Zhang, Jingquan Liu, Jianming Zhou

**Affiliations:** 1 Department of Critical Care Medicine, Hangzhou TCM Hospital Affiliated to Zhejiang Chinese Medical University, No.453, Tiyuchang Road, Hangzhou, 310007 Zhejiang China; 2 Department of Critical Care Medicine, Zhejiang Provincial People’s Hospital, Hangzhou, China

**Keywords:** Cerebral infarction, Lactylation, LCP1, Glycolysis

## Abstract

Cerebral infarction (CI), also known as ischemic stroke, has a high incidence rate and mortality rate. The purpose of this study was to investigate the potential effect and mechanism of Lymphocyte cytosolic protein 1 (LCP1) in the CI progression. The middle cerebral artery occlusion (MCAO) treated rats and oxygen–glucose deprivation/reoxygenation (OGD/R) stimulated PC12 cells were used to establish CI model in vivo and in vitro. The cell proliferation and apoptosis was determined by CCK-8 assay and flow cytometry, respectively. Immunoprecipitation and western blot was performed to test the lactylation levels of LCP1. The cells were treated with cycloheximide to determined the protein stability of LCP1. The glucose uptake and lactate production was determined with commercial kits. The extracellular acidification rate were evaluated by Seahorse. The results showed that LCP1 was upregulated in the MCAO rats and OGD/R stimulated PC12 cells. LCP1 knockdown dramatically decreased the neurological score, infarct volume and the brain water content of MCAO rats. Besides, LCP1 knockdown promoted the cell viability while decreased the apoptosis rate of the OGD/R stimulated PC12 cells. Additionally, the global lactylation and lactylation levels of LCP1 was prominently enhanced in vivo and in vitro in cerebral infarction. 2-DG treatment prominently decreased it. In conclusion, inhibiting the glycolysis decreased the lactylation levels of LCP1 and resulted in the degradation of LCP1, which eventually relieved the CI progression.

## Introduction

Cerebral infarction (CI) has a high incidence rate and mortality rate [1]. After CI, cerebral blood circulation is impaired, ischemia and hypoxia damage occurs in local tissues, which often leads to neuronal damage, and finally leads to paralysis, aphasia and other neurological deficit symptoms [2–4]. Thus, effective methods to alleviate neuronal damage after brain injury is of great significance to promote the recovery of neurological function in patients with cerebral infarction.

As an actin-binding protein, Lymphocyte cytosolic protein 1 (LCP1) regulates cell movement mainly by interacting with actin, and is one of the main cytoskeleton binding proteins that directly control cell movement [5]. In addition, LCP1 is also involved in host defense, the formation of immune synapses, T cell activation, etc. [6]. Under normal circumstances, LCP1 is mainly expressed in hematopoietic cells, but it is also highly expressed in a variety of malignant tumor cells [7]. The high expression of LCP1 in tumor cells is closely related to tumor invasion and metastasis, and its abnormal expression can be used as an effective prognosis marker for tumors [8–10]. Recently, through the global proteomic analysis, wen et al. [11] found that LCP1 was one of the upregulated genes in the ischemia–reperfusion (I/R) injury model, and fold change is greater than 2 at 14 days in I/R. However, the function and precise mechanism of LCP1 in CI remains unclear.

Nowadays, increasing studies have shown that abnormal energy metabolism is closely related to many diseases such as diabetes, cancer and CI [12–14]. It has been reported that under normal conditions, neurons have lower levels of glycolysis, but when CI occurs, the aerobic respiration of neurons will be inhibited, and the glycolysis promoted to maintain energy supply [15, 16]. However, during the reperfusion period of blood oxygen recovery, the metabolism of neurons is still in a high glycolysis state. This phenomenon is called the "Warburg effect". Even if oxygen is sufficient, neurons will still preferentially choose glycolysis for energy supply, which will further aggravate the death of neurons [17, 18]. As the end product of glycolysis, lactate was considered to be the metabolic waste without critical regulatory functions. However, recent studies uncovered a novel function of glycolysis-derived lactate in macrophages, to regulate histones modification by adding lactate groups to lysine residues, which is called lactylation [19]. However, the mechanism of lactylation in CI progression is less studied, and whether lactate can promote the lactylation and expression of LCP1 is unclear.

Therefore, this study aimed to explore the role of LCP1 in CI progression through the in vivo and in vitro studies. Moreover, we tried to demonstrate the regulatory role of lactate on LCP1 expression. We hope these findings will provide novel targets for the treatment of cerebral ischemia diseases.

## Materials and Methods

### Animal Experiment

Eighteen healthy adult male Sprague Dawley (SD) rats weighing about 250–300 g were purchased from Beijing Vital River Laboratory Animal Technology Co., Ltd. (Beijing, China). Feeding conditions was as follows: The temperature is constant at (22 ± 1)°C, the humidity is (50 ± 20)%, and the light conditions are 12 h day and night, all the rats were free access to the food and water. Animal experiments were approved by the experimental animal ethics committee of the hospital. After adaptive feeding, the middle cerebral artery occlusion (MCAO) was performed to establish a CI rat model. The rats were anesthetized by intraperitoneal injection of 1% sodium pentobarbital (50 mg/kg). All rats were dissected in the middle of the neck. The left external carotid artery (ECA) was exposed and dissected. A monofilament nylon was inserted from ECA into the internal carotid artery to block the left middle cerebral artery. After 90 min of ischemia, the monofilament nylon was removed. Then, ECA wound was sutured and reperfusion was performed for 24 h. Rats in the sham group were subjected to the same operations except for suffering from monofilament obstruction. For LCP1 knockdown, the rats were injected with 1 μL Lentivirus silencing LCP1 (LV-sh-LCP1) and LV-sh-nc at the sites of the cortex and hippocampus 14 days before modeling. After 24 h of cerebral ischemia–reperfusion, the neurological function and behavior was observed. Then the rats were sacrificed after anesthesia, and the brain tissue was collected.

### Neurological Deficit Score

The rats were placed on the ground, and the limb movements were observed. The neurological score of each group were scored according to the Longa method [20]. Normal activity and asymptomatic were scored as 0 points, the left forelimb could not be fully extended as 1 point, crawling resistance decreased when pushing to the left, 2 points, turning to the left when crawling was 3 points, and unconsciousness and inability to walk were scored as 4 points.

### Triphenyl Tetrazolium Chloride (TTC) Staining Assay

The brain tissues were stored at − 20 °C for 20 min and then cut into 1 mm pieces. Then the sections were incubated with 1% TTC solution (Senjbio, Nanjing, China) at 37 °C for 20 min. Next, the sections were fixed with 4% paraformaldehyde and the infarct volume was calculated using the Image Pro Plus6.0 Image pro Plus6.0 image analysis system. Non-infarcted myocardium is red and ischemic necrotic tissue is white. Infarct volume was quantified.

### Brain Water Content

The brain water content was determined using wet/dry method. The wet weight of ischemic brain tissues (right) was weighed. Then the tissues were incubated in an oven at 110 °C for 24 h. After that, the dry weight of the ischemic brain tissues (right) was re- weighed. The brain water content was calculated using the formula:$\left( {{\text{wet weight}} - {\text{dry weight}}} \right)/{\text{wet weight}} \times {1}00\% .$

### Cell Culture and Treatment

Neurocytes (PC12) were purchased from ATCC (Manassas, USA) and grown in DMEM (Gibco, Grand Island, USA) supplemented with 10% FBS and 1% penicillin/streptomycin. The cells were cultured at 37 °C with 5% CO_2_. For OGD/R treatment, the cells were exposed to glucose free medium and then placed in a modular chamber containing mixed gas (37 °C, 5% CO_2_, and 94% N_2_,). After 2 h, the cells were replaced with normal medium and cultured under normal oxygen. Cells cultured under normal conditions served as control group. Additionally, normal PC12 cells were treated with 25 mM of Lactate for 8, 16, 24 h to induced the glycolysis. OGD/R stimulated PC12 cells were treated 20 mM 2-DG for 24 h to inhibited the glycolysis. The cell growth was observed using the light microscope.

### Cell Transfection

The short hairpin RNA targeting LCP1 (sh-LCP1) and sh-NC was synthesized by Genechem Biological company (Shanghai, China) and transfected into the cells using Lipofectamine 3000 (Invitrogen, Carlsbad, USA). After 48 h, the transfected cells were collected for the next experiments.

### Measurement of Glycolysis

Extracellular acidification rate (ECAR) were measured as previously described [21]. Briefly, the cells were incubated in 96-well plates. For ECAR detecting, glucose was added at 10 min, oligomycin was added at 40 min, and 2-deoxy-d-glucose (2-DG) was added at 70 min using Seahorse auto fill. ECAR was detected using a Seahorse XF glycolysis stress test kit (Agilent, Beijing) from 0 to 100 min following the manufacturer’s protocol. The results were measured using Seahorse XF96 Analyzer (Agilent). Additionally, the glucose uptake and lactate production was determined with Glucose Uptake Assay Kit (Agilent) and Lactate Assay Kit (Agilent). All operations shall be carried out in strict accordance with the instructions of the kits.

### Cell Viability Detection

Cell counting kit-8 kit (Dojindo, Kumamoto, Japan) was utilized to measure the cell viability. Transfected cells were seeded into 96-well plates and incubated at 37 °C for 24 h. Subsequently, 10 μL CCK-8 solution was used to incubate with cells for 2 h. Absorbance was detected using a microplate reader (Bio-Rad, Hercules, CA) at 450 nm.

### Determination of Cell Apoptosis

Apoptosis was evaluated using Annexin V-PE/7AAD kit (Solarbio). The cell suspension was incubated with 5 μL Annexin V/PE for 5 min, followed by incubation with 10 μL 7AAD. After adding 400 μL PBS, apoptosis was detected using a flow cytometer (Beckman Coulter, Miami, USA).

### Determination of Energy Metabolism

The adenosine-triphosphate (ATP), adenosine diphosphate (ADP), adenosine monophosphate (AMP), complex I, and complex IV levels were detected using corresponding kits, which were purchased from the Sigma (CA, USA). All operations shall be carried out according to the instructions of the kit. The energy charge (EC) was calculated using the following formula:

$${\text{EC}} = \left( {{\text{ATP}} + {1}/{\text{2ADP}}} \right)/\left( {{\text{ATP}} + {\text{ADP}} + {\text{AMP}}} \right).$$

### Western Blotting

Cells or tissues were incubated with RIPA buffer (Beyotime, Shanghai, China) for cell lysis. The protein content was measured using a BCA protein assay kit (Beyotime). The proteins (30 μg/lane) were run on SDS-PAGE and transferred to PVDF membranes. Then, 5% skim milk was added to block the membranes for 2 h. The membranes were incubated with primary antibodies against LCP1 (Abcam), pan-kla (PTM Bio) or GAPDH (Abcam) at 4 °C overnight, followed by incubating with secondary antibody at room temperature for 2 h. The protein bands were shown using an ECL substrate (Solarbio). GAPDH was used as the internal control.

For the determination of LCP1 protein stability, the cells were treated with cycloheximide (CHX) to inhibit protein synthesis. The protein levels of LCP1 after CHX treatment for 0, 2, 4, 8 h were detected by western blot. According to the signal intensity of protein bands, the relative expression of each protein was calculated and the half-life curve of LCP1 protein was drawn.

### Immunoprecipitation (IP)

The protein lysates were treated with NP40 lysis buffer (1 mM PMSF and 1 mM DTT). Then the protein lysates wee incubated with Add 1 μg primary antibody LCP1 on a shaker at 4 °C overnight. On the second day, each EP tube was added with 20 μL protein A/G beads, and incubated for 2 h at 4 °C. Next, the IP tube was centrifuged at 4 °C, 1000 rpm for 1 min, After washing, the beads were boiled and separated on the SDS-PAGE electrophoresis. Then, the western blot was performed for the lactylation level of LCP.

### Statistical Analysis

Data are presented as mean ± standard deviation using GraphPad Prism 8 software (GraphPad, La Jolla, CA). Differences were assessed using Student’s *T*-test or one way ANOVA. *p* < 0.05 was considered as statistically significant.

## Results

### LCP1 was Upregulated in the Brain Tissues of MCAO Rats

**Fig. 1:**
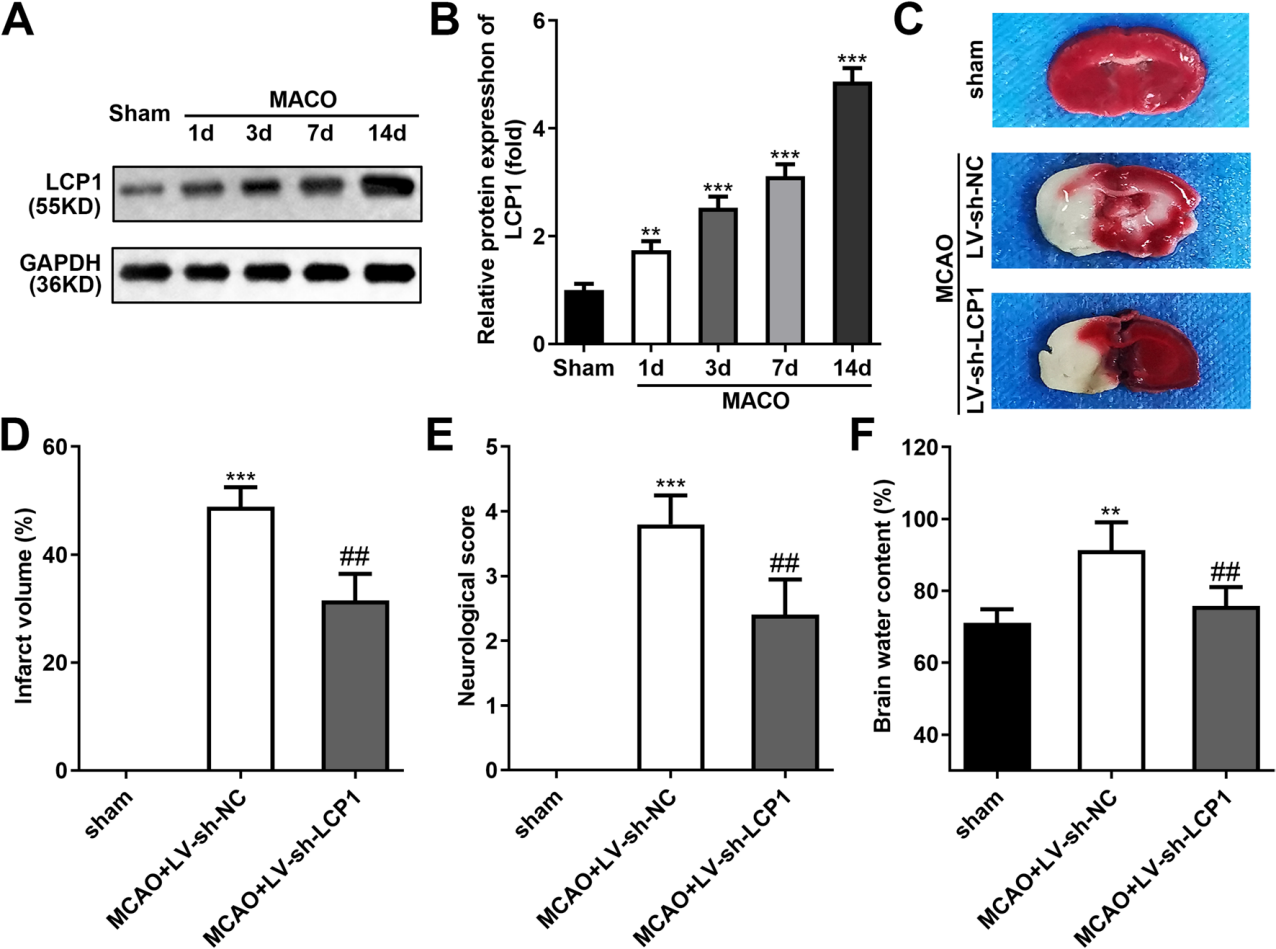
LCP1 was upregulated in the brain tissues of MCAO rats. **A**–**B** Protein levels of LCP1 in the brain tissues of MCAO rats at 1, 3, 7, 14 days. **C**–**D** TTC staining and the results of infarct volume of brain tissues in MCAO rats treated with LV-sh-LCP1. The neurological score (**E**) and brain water content (**F**) of brain tissues in MCAO rats treated with LV-sh-LCP1. **P < 0.01, ***P < 0.001 versus sham group. ^##^*P* < 0.01 vs MCAO + LV-sh-NC group

The protein levels of LCP1 were dramatically upregulated in the brain tissues of MCAO rats, and the increase of LCP was time-dependent (Fig. 1A–B). The TTC staining showed that the infarct volume was prominently elevated in the brain tissues of MCAO rats, while LCP1 knockdown dramatically declined it (Fig. 1C–D). Besides, the neurological score (Fig. 1E) and brain water content (Fig. 1F) was dramatically elevated in the brain tissues of MCAO rats, and LCP1 knockdown dramatically declined them.

### LCP1 was Upregulated in the OGD/R Stimulated PC12 Cells

**Fig. 2:**
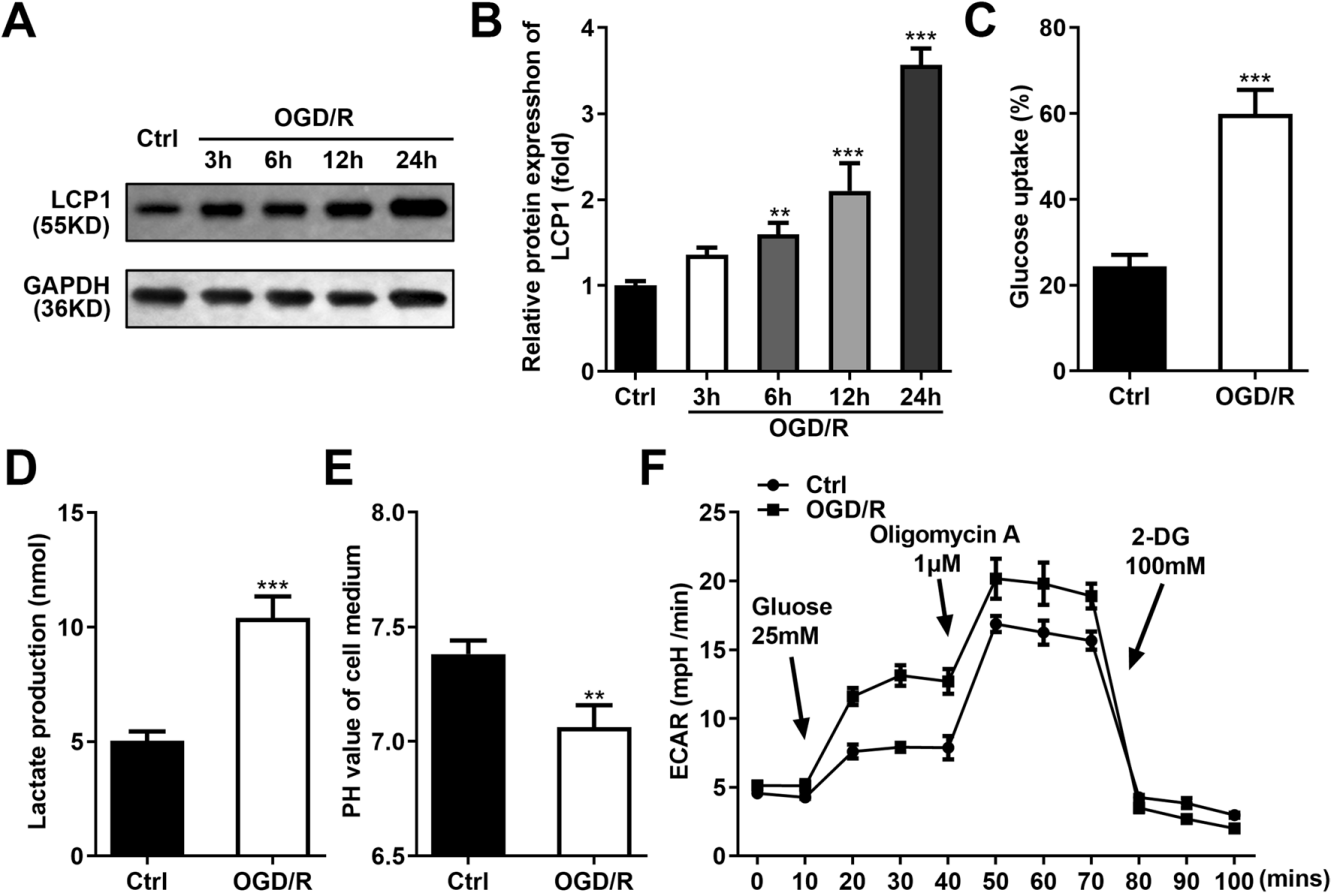
LCP1 was upregulated in the OGD/R stimulated PC12 cells. **A**–**B** Protein levels of LCP1 in the OGD/R stimulated PC12 cell at 3, 6, 12, 24 h. Glucose uptake (**C**), lactate production (**D**), PH value of cell medium (**E**) and ECAR (**F**) levels in the PC12 cells was determined after OGD/R stimulation. **P < 0.01, ***P < 0.001 versus ctrl group

After OGD/R stimulation, the protein levels of LCP1 were dramatically upregulated in the PC12 cells, and the increase of LCP was time-dependent (Fig. 2A–B). Additionally, OGD/R stimulation prominently elevated the glucose uptake (Fig. 2C), lactate production (Fig. 2D), and declined the PH value of cell medium (Fig. 2E). Furthermore, OGD/R stimulation prominently elevated the ECAR of the PC12 cells (Fig. 2F). These results suggested OGD/R stimulation promoted glycolysis.

### LCP1 Knockdown Prevented the Cell Death in the OGD/R Stimulated PC12 Cells

**Fig. 3:**
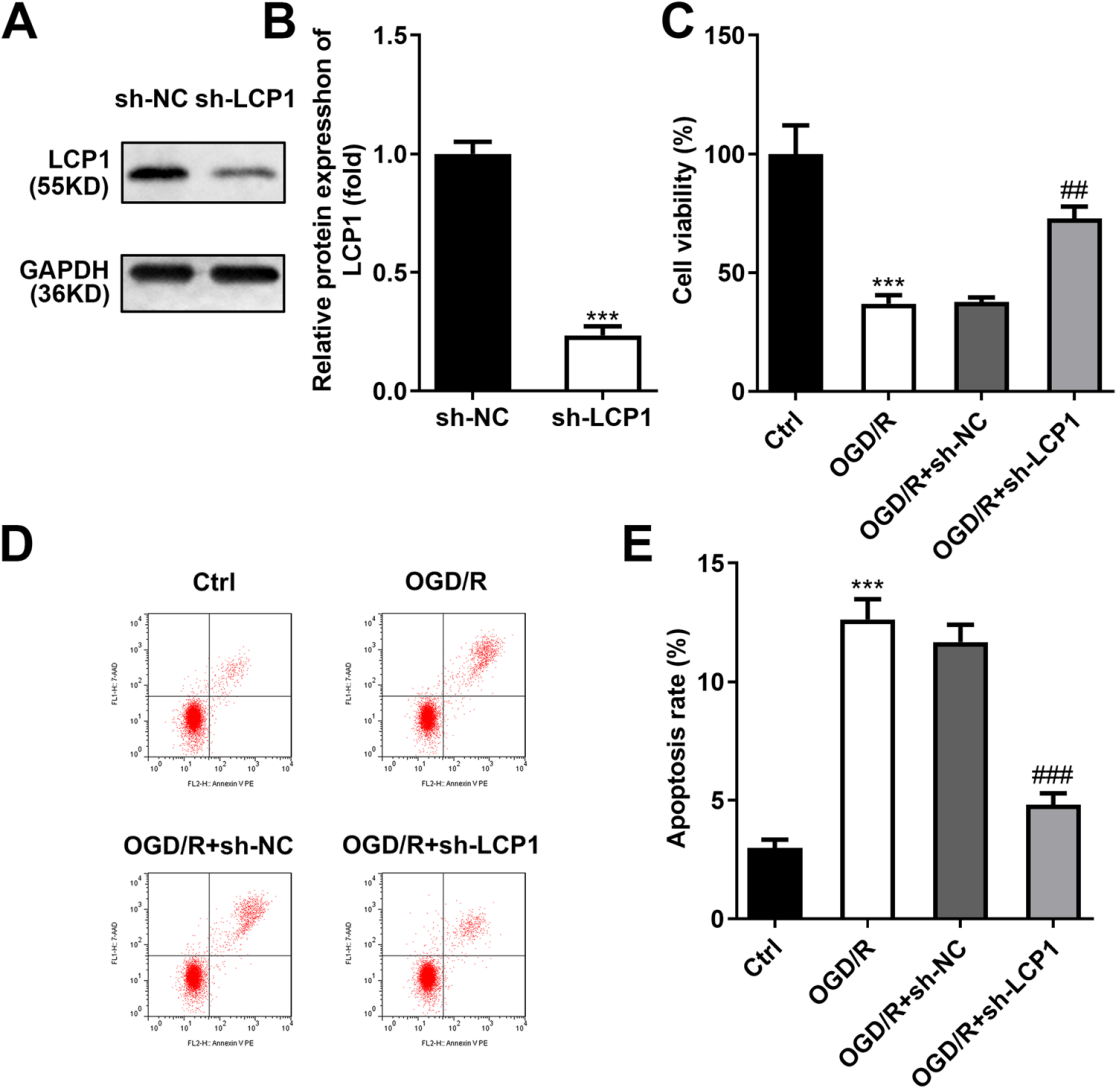
LCP1 knockdown prevented the cell death in the OGD/R stimulated PC12 cells. **A**–**B** The transfection efficiency of sh-LCP1 was verified by western blot. The OGD/R stimulated PC12 cells were transfected with sh-LCP1. **C** Cell viability was assessed with CCK-8 assay. **D**–**E** Apoptosis rate was assessed with flow cytometry. ***P < 0.001 versus ctrl group. ^##^*P* < 0.01, ^###^*P* < 0.001 versus OGD/R + sh-NC group

The transfection efficiency of sh-LCP1 was verified by western blot, which showed that sh-LCP1 dramatically declined the LCP1 levels in the PC12 cells (Fig. 3A–B). Furthermore, OGD/R stimulation prominently decreased the cell viability (Fig. 3C) while promoted the apoptosis (Fig. 3D–E) of the PC12 cells. LCP1 knockdown prominently elevated the cell viability and prevented the apoptosis in the OGD/R stimulated PC12 cells.

### LCP1 Knockdown Inhibited the Energy Metabolism in the OGD/R Stimulated PC12 Cells

**Fig. 4:**
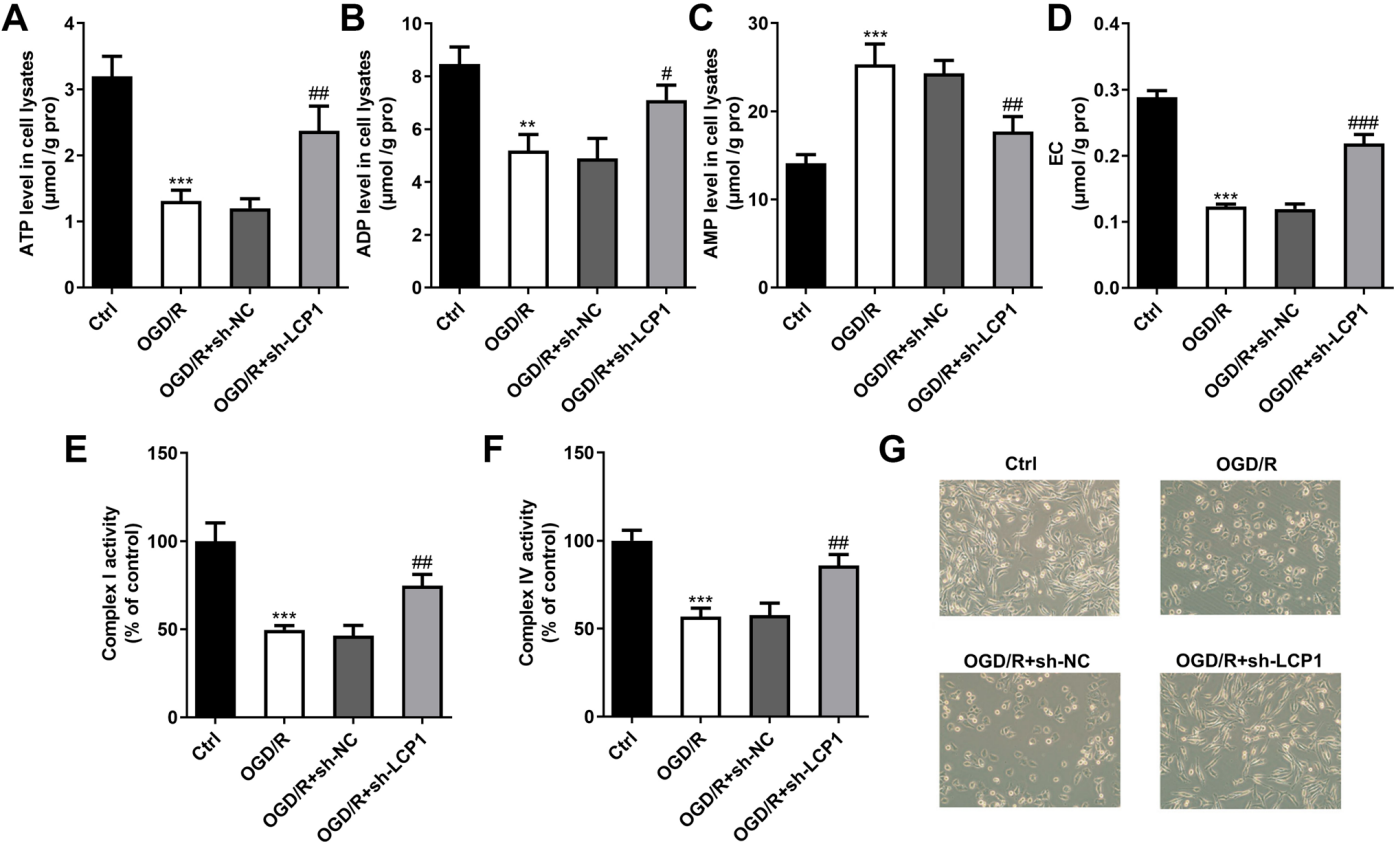
LCP1 knockdown inhibited the energy metabolism in the OGD/R stimulated PC12 cells. The OGD/R stimulated PC12 cells were transfected with sh-LCP1. The ATP (**A**), ADP (**B**), AMP (**C**), EC (**D**), complex I (**E**) and complex IV (**F**) levels were detected using corresponding kits. **G** The cell growth was observed using light microscope. ***P < 0.001 versus ctrl group. ^#^*P* < 0.05, ^##^*P* < 0.01 versus OGD/R + sh-NC group

Subsequently, we found that OGD/R treatment significantly decreased the ATP (Fig. 4A), ADP (Fig. 4B) and EC (Fig. 4D) levels, and increased the AMP (Fig. 4C) levels in the PC12 cells. After LCP1 knockdown, the ATP, ADP and EC levels in the OGD/R stimulated PC12 cells were increased, and AMP levels were decreased. Besides, we found that OGD/R treatment significantly decreased complex I (Fig. 4E) and complex IV (Fig. 4F) activities in the PC12 cells, while LCP1 knockdown enhanced them. Additionally, through the light microscope observation, we found that OGD/R treatment significantly inhibited the cell growth of PC12 cells and LCP1 knockdown enhanced it (Fig. 4G).

### OGD/R Stimulation Enhanced the Lactylation Levels of the PC12 Cells

**Fig. 5:**
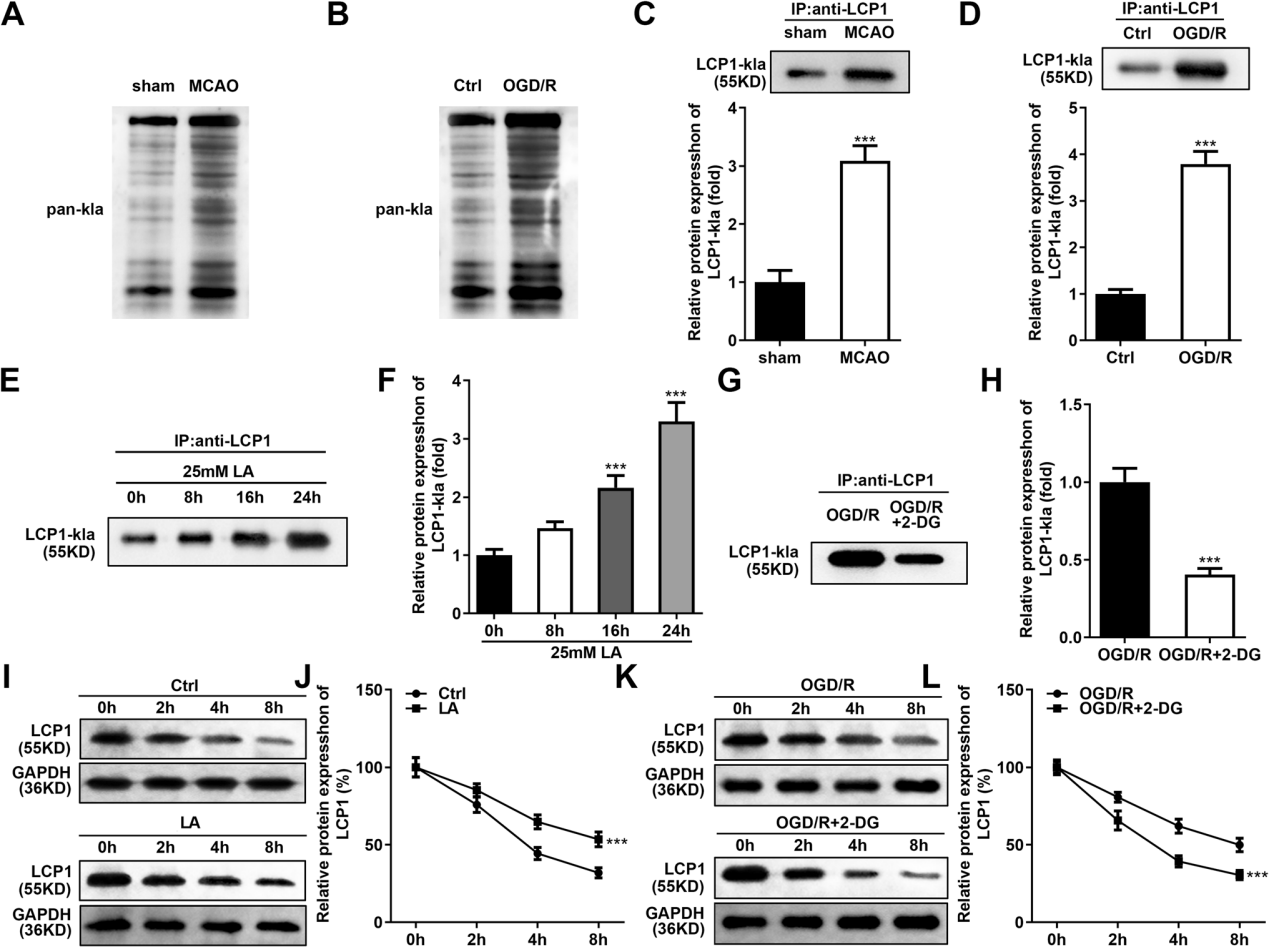
OGD/R stimulation enhanced the lactylation levels of the PC12 cells. The global lactylation levels in the MCAO rats (**A**) and OGD/R stimulated PC12 cells (**B**). The lactylation levels of LCP1 in the MCAO rats (**C**) and OGD/R stimulated PC12 cells (**D**). **E**–**F** The lactylation levels of LCP1 in the LA-treated PC12 cells. **G**–**H** The lactylation levels of LCP1 in the OGD/R stimulated PC12 cells treated with 2-DG. ***P < 0.001

Hypoxia induction will lead to the increase of lactic acid level. Therefore, we speculated that whether OGD/R stimulation will lead to lactylation in the PC12 cells. The western blot results showed that the global lactylation was prominently enhanced in the MCAO rats (Fig. 5A) and OGD/R stimulated PC12 cells (Fig. 5B). Then, we analyzed the lactylation levels of LCP1 through the IP and western blot assays. The results showed that lactylation levels of LCP1 were prominently elevated in the MCAO rats (Fig. 5C) and OGD/R stimulated PC12 cells (Fig. 5D). Then, after LA treatment, the lactylation levels of LCP1 were prominently upregulated in the PC12 cells and the increase was time-dependent (Fig. 5E–F). After 2-DG treatment, the lactylation levels of LCP1 were prominently downregulated in the OGD/R stimulated PC12 cells (Fig. 5G–H). Next, through the CHX treatment, the degradation of LCP1 was inhibited in the LA-treated PC12 cells (Fig. 5I–J). Additionally, the degradation of LCP1 was accelerated in the OGD/R stimulated PC12 cells treated with 2-DG (Fig. 5K–L).

### Inhibition of the Glycolysis Attenuated the Cell Apoptosis in the OGD/R Stimulated PC12 Cells

**Fig. 6:**
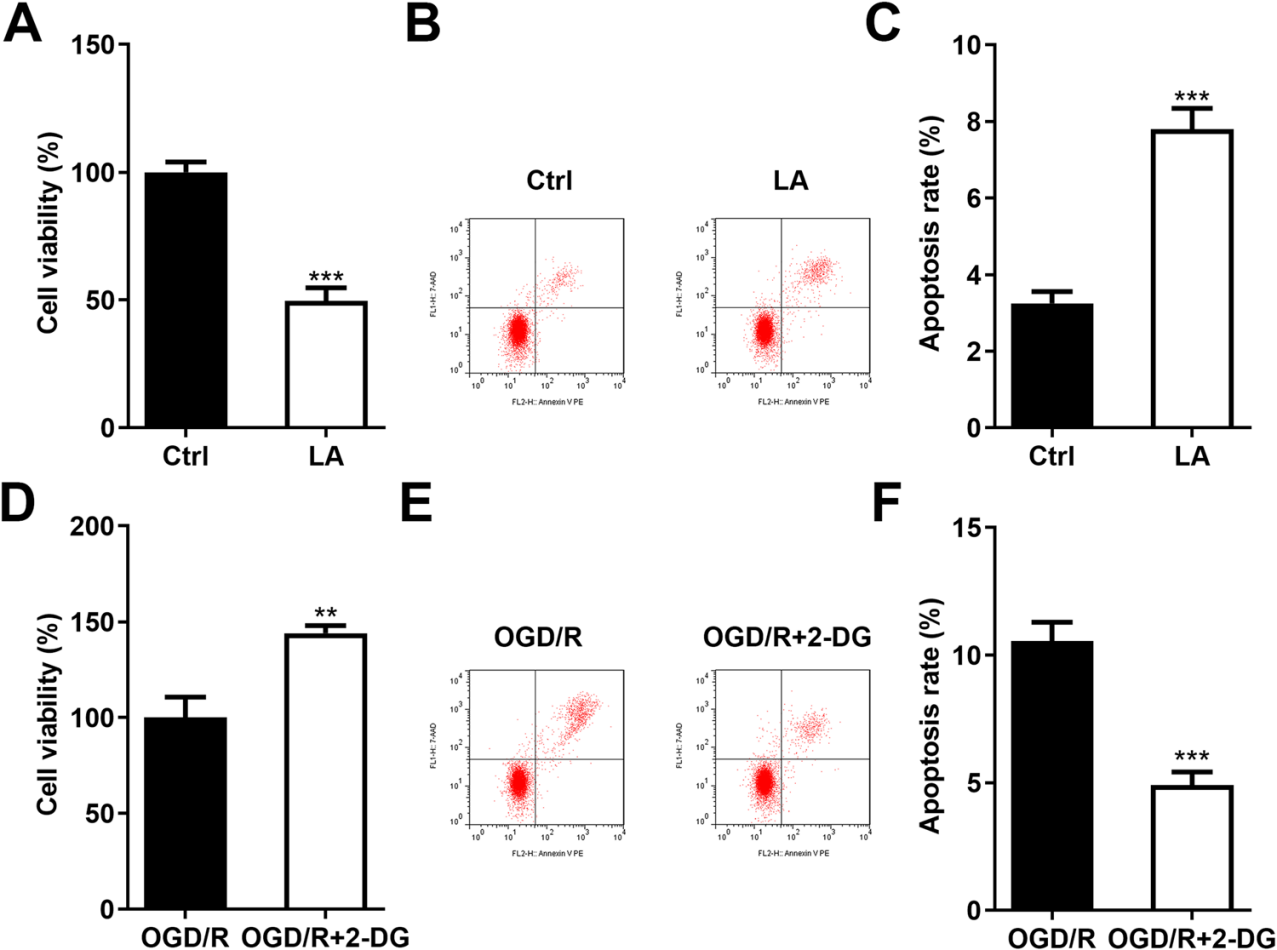
Inhibition of the glycolysis inhibited the cell apoptosis in the OGD/R stimulated PC12 cells. **A** The cell viability of the PC12 cells was assessed using CCK-8 assay after LA treatment. **B**–**C** The apoptosis rate of the PC12 cells was assessed using flow cytometry after LA treatment. **D** The cell viability of the OGD/R stimulated PC12 cells was assessed using CCK-8 assay after 2-DG treatment. **E**–**F** The apoptosis rate of the OGD/R stimulated PC12 cells was assessed using flow cytometry after 2-DG treatment. **P < 0.01, ***P < 0.001

After LA treatment, the cell viability (Fig. 6A) was prominently declined and the apoptosis rate (Fig. 6B–C) was prominently enhanced in the PC12 cells. Besides, after 2-DG treatment, the cell viability (Fig. 6D) was prominently enhanced and the apoptosis rate (Fig. 6E–F) was prominently decreased in the PC12 cells.

## Discussion

In the current study, we demonstrated that LCP1 was highly expressed in the MCAO rats and OGD/R stimulated PC12 cells. Depletion of LCP1 relieved the injury in the MCAO rats and OGD/R stimulated PC12 cells. Additionally, the global lactylation and lactylation levels of LCP1 were elevated in vivo and in vitro. Inhibition of glycolysis with 2-DG decreased the lactylation levels of LCP1 and relieved the injury of PC12 cells induced by OGD/R.

Recently, three isoforms of plastin, which comprises actin-binding proteins, were demonstrated to exist in mammals [22]. Among them, LCP1 was expressed in hematopoietic cellular lineages and many types of cancers, which regulated the cell movement by interacting with actin and plays a key role in the process of cell migration [9]. At present, the functional research of LCP1 is mainly focused on cancer. The abnormally expressed LCP1 is closely related to the stages and severity of various cancers, and LCP1 has been regarded as a potential prognostic indicator in colon and breast cancers [23, 24]. Ning et al. found that LCP1 was upregulated and acted as a specific molecular marker of cancer recurrence and therapeutic targets in colon cancer [25]. Similarly, LCP1 was confirmed to promote the cell invasion and metastasis in prostate cancer cells, and the silencing experiment indicated that LCP1 might be an effective approach for tumors treatment [5, 22]. On the other hand, Wen et al. [11] performed a global proteomic analysis to explore the differently expressed genes in the cerebral ischemia–reperfusion injury through MCAO rat model. They confirmed that LCP1 was dramatically up-regulated in model group at 14 days. This result attracted my attention. We speculated that LCP1 might be a potential target for the treatment of CI. In this study, we found that LCP1 was over expressed in the MCAO rats and OGD/R stimulated PC12 cells. Depletion of LCP1 promoted the cell growth of OGD/R stimulated PC12 cells and relieved the injury in the MCAO rats. These results preliminarily explained the promoting effect of PC12 on the development of CI.

The study found that after craniocerebral injury, the blood cerebrospinal fluid barrier was destroyed, the vascular permeability increased, and the metabolism of Lactate was abnormal and accumulated in blood [26]. Lactate is an important carbon containing metabolite in the glycolysis pathway of cells. Its biological function has received extensive attention due to the existence of Warburg effect. Lactylation is a post-translational modification type of protein reported by Zhao et al. [19]. Subsequent studies further confirmed that protein lactylation is an important way for lactate to exert biological functions, and is demonstrated wo be involved in many life activities such as uterine remodeling [27], tumor proliferation [28], nervous system regulation [29], metabolic regulation [30]. Here, we found that the lactate production and glucose uptake was prominently enhanced in the OGD/R stimulated PC12 cells, which This led us to speculate that whether the excessive production of lactate would lead to an increase of lactylation. Interestingly, we found that the global lactylation and lactylation levels of LCP1 were elevated in vivo and in vitro. Subsequently, the lactate treated PC12 cells showed the same results on the lactylation levels of LCP1 compared with the OGD/R stimulated PC12 cells and lactate treatment elevated the protein stability of LCP1. These findings implied that LCP1 promoted the CI progression through the lactate mediated lactylation modification. Importantly, after 2-DG treatment, a glycolysis inhibitor, the lactylation levels and stability of the LCP1 was significantly declined. All these results indicated that the high levels and stability of LCP1 in the CI progression were induced by the lactate mediated lactylation modification.

In conclusion, this study explored the effects of lactylation on the LCP1 in CI for the first time. We demonstrated that lactylation promoted the stability of LCP1, which further promoted the CI development. This study revealed the role of lactylation modified LCP1 in the occurrence and development of CI, and provided a new vision for the treatment of CI in the future.

## Data Availability

The datasets used and analyzed during the current study are available from the corresponding author on reasonable request.
